# Clinical Significance of Organic Anion Transporting Polypeptide Gene Expression in High-Grade Serous Ovarian Cancer

**DOI:** 10.3389/fphar.2018.00842

**Published:** 2018-08-07

**Authors:** Martin Svoboda, Felicitas Mungenast, Andreas Gleiss, Ignace Vergote, Adriaan Vanderstichele, Jalid Sehouli, Elena Braicu, Sven Mahner, Walter Jäger, Diana Mechtcheriakova, Dan Cacsire-Tong, Robert Zeillinger, Theresia Thalhammer, Dietmar Pils

**Affiliations:** ^1^Department of Pathophysiology and Allergy Research, Center for Pathophysiology, Infectiology and Immunology, Medical University of Vienna, Vienna, Austria; ^2^Institute of Clinical Biometrics, Center for Medical Statistics, Informatics, and Intelligent Systems, Medical University of Vienna, Vienna, Austria; ^3^Division of Gynaecological Oncology, Department of Gynaecology and Obstetrics, Leuven Cancer Institute, University Hospital Leuven, Katholieke Universiteit Leuven, Leuven, Belgium; ^4^Department of Gynecology, Charité – Universitätsmedizin Berlin, Corporate Member of Freie Universität Berlin, Berlin Institute of Health, Humboldt-Universität zu Berlin, Berlin, Germany; ^5^Department of Gynecology, University Medical Center Hamburg-Eppendorf, Hamburg, Germany; ^6^Department of Clinical Pharmacy and Diagnostics, University of Vienna, Vienna, Austria; ^7^Translational Gynecology Group, Department of Obstetrics and Gynaecology, Comprehensive Cancer Center, Medical University of Vienna, Vienna, Austria; ^8^Molecular Oncology Group, Department of Obstetrics and Gynaecology, Comprehensive Cancer Center, Medical University of Vienna, Vienna, Austria; ^9^Department of Surgery, Medical University of Vienna, Vienna, Austria

**Keywords:** high-grade serous ovarian cancer, *SLCO*, OATP, transporter, overall survival, estrogens

## Abstract

High-grade serous ovarian cancer (HGSOC) is considered the most deadly and frequently occurring type of ovarian cancer and is associated with various molecular compositions and growth patterns. Evaluating the mRNA expression pattern of the organic anion transporters (OATPs) encoded by *SLCO* genes may allow for improved stratification of HGSOC patients for targeted invention. The expression of *SLCO* mRNA and genes coding for putative functionally related ABC-efflux pumps, enzymes, pregnane-X-receptor, *ESR1* and *ESR2* (coding for estrogen receptors ERα and ERß) and HER-2 were assessed using RT-qPCR. The expression levels were assessed in a cohort of 135 HGSOC patients to elucidate the independent impact of the expression pattern on the overall survival (OS). For identification of putative regulatory networks, Graphical Gaussian Models were constructed from the expression data with a tuning parameter K varying between meaningful borders (Pils et al., 2012; Auer et al., 2015, 2017; Kurman and Shih Ie, 2016; Karam et al., 2017; Labidi-Galy et al., 2017; Salomon-Perzynski et al., 2017; Sukhbaatar et al., 2017). The final value used (*K* = 4) was determined by maximizing the proportion of explained variation of the corresponding LASSO Cox regression model for OS. The following two networks of directly correlated genes were identified: (i) *SLCO2B1* with *ABCC3* implicated in estrogen homeostasis; and (ii) two ABC-efflux pumps in the immune regulation (*ABCB2/ABCB3*) with *ABCC3* and *HER-2*. Combining LASSO Cox regression and univariate Cox regression analyses, *SLCO5A1* coding for OATP5A1, an estrogen metabolite transporter located in the cytoplasm and plasma membranes of ovarian cancer cells, was identified as significant and independent prognostic factor for OS (HR = 0.68, CI 0.49–0.93; *p* = 0.031). Furthermore, results indicated the benefits of patients with high expression by adding 5.1% to the 12.8% of the proportion of explained variation (PEV) for clinicopathological parameters known for prognostic significance (FIGO stage, age and residual tumor after debulking). Additionally, overlap with previously described signatures that indicated a more favorable prognosis for ovarian cancer patients was shown for *SLCO5A1*, the network *ABCB2/ABCB3/ABCC4/HER2* as well as *ESR1*. Furthermore, expression of *SLCO2A1* and *PGDH*, which are important for PGE_2_ degradation, was associated with the non-miliary peritoneal tumor spreading. In conclusion, the present findings suggested that *SLCOs* and the related molecules identified as potential biomarkers in HGSOC may be useful for the development of novel therapeutic strategies.

## Introduction

High-grade serous ovarian cancer (HGSOC) is the most frequent occurring and aggressive subtype of all ovarian cancer types. HGSOC is often diagnosed late and is associated with therapeutic resistance following surgical debulking and platin- and taxane-based chemotherapy as standard of care. These factors contribute to the high mortality. Since the poor overall survival (OS) of HGSOC patients has not improved greatly during the last decades, more knowledge on the characteristic of these tumors is required (Karam et al., 2017).

HGSOC develops from epithelial cells in the fimbriae region of the fallopian tubes that undergo neoplastic transformation to serous tubal intraepithelial carcinomas (STICs) to form HGSOC in the ovary (Labidi-Galy et al., 2017). In some cases, HGSOC might also develop from the less aggressive low-grade serous ovarian carcinomas (LGSOC), which arises from ovarian tissue (Auer et al., 2017; Sukhbaatar et al., 2017). Notably, mutations in *TP53* are considered important for the diagnosis of HGSOC and are observed in >95% of cases (Kurman and Shih Ie, 2016). Aside from *TP53* mutations, HGSOC can also present with variable molecular compositions and growth patterns, thus a further sub-classification based on the morphologic and molecular heterogeneity of these tumors is recommended (Pils et al., 2012; Salomon-Perzynski et al., 2017).

In contrast to other cancer types of the reproductive organs with broad lymphatic or haematogenous dissemination to distant organs, including cervix and breast, ovarian cancer cells spread predominantly in the peritoneal cavity. Of note, epithelial and mesenchymal markers are simultaneously expressed in HGSOC cells and a continuous dynamic epithelial/mesenchymal transition (EMT) can enable tumor cells to adapt for peritoneal implantation and local tumor metastasis (Auer et al., 2015). It is thought that tumor cells in the peritoneal fluid and those circulating in the blood implant in the peritoneum and omentum to form metastatic tumors, which can result in the destruction of neighboring organs (Yeung et al., 2015; Obermayr et al., 2017). A close relation between the pattern of peritoneal metastases and the putative origin of HGSOC tumor cells in the fallopian tubes (T) or the ovary (O) was previously demonstrated by a comparative transcriptome analyses (Pradeep et al., 2014; Auer et al., 2015; Bachmayr-Heyda et al., 2017). Tumor cells of ovarian origin grow in the peritoneum in a non-miliary (n-M) fashion and result in the production of large tumor nodes. Typically these n-M tumors can be removed completely, which contributes to improved prognosis of tumors with this pattern of metastasis. However, miliary (M) growing tumors form many small (millet-sized) tumor nodes, which at the time of the diagnosis are widely spread in the peritoneum. According to the signature of M tumors, they originate from the fallopian tube epithelium. Because complete removal is usually difficult to achieve and they seem to be more aggressive than other ovarian cancers, the prognosis is generally worse than that of n-M tumors (Pils et al., 2012; Torres et al., 2017).

Other classification systems to stratify HGSOC patients according to their risk for a cancer-related death were developed as molecular signatures for these tumors. Similar to breast cancer, many tumors express estrogen receptor alpha (ERα), estrogen receptor beta (ERβ) and/or other steroid hormone receptors. A subgroup of these tumors express additionally/or exclusively epidermal growth factor receptor 2 (HER-2, ERBB2); however, some tumors been demonstrated to express none of these markers. Importantly, HGSOC tumors expressing high levels of ERα are usually sensitive to estrogens and are associated with a better prognosis compared with tumors expressing HER-2, but lack expression of ERα (Voutsadakis, 2016; Rižner et al., 2017). Another risk classification system to stratify HGSOC patients into two groups (group 1 and group 2) based on molecular signature was defined by Yoshihara (Yoshihara et al., 2009). Group 1 is characterized by a better response to chemotherapy and a more favorable prognosis compared to group 2. Most prominent in this identified signature is the reduced expression of immune-response-related genes in tumors classified into group 2. Furthermore, genes coding for transport proteins in the antigen presentation pathway are downregulated. The ATP-binding cassette (ABC)-transporters ABCB2 (TAP1) and ABCB3 (TAP2), which are encoded by *ABCB2* and *ABCB3* genes, respectively, are part of the major histocompatibility complex class I restricted antigen processing machinery. Consequently, high levels of *ABCB2/ABCB3* are associated with improved survival in ovarian cancer patients, indicating the importance of transport proteins in ovarian cancer progression.

Aside from the immune system, many transmembrane transport proteins play an important role in cancer cell survival and progression in relation to their transport function in cancer cells (Thakkar et al., 2015). For uptake of drugs, xenobiotics and compound from the endogenous metabolism, eleven member of the family of organic anion transporting polypeptides (OATPs) encoded by *SLCO* genes have been identified (Hagenbuch and Gui, 2008; Obaidat et al., 2012; Stieger and Hagenbuch, 2014). Eleven members of the organic anion transporting polypeptides (OATPs) family encoded by *SLCO* genes have been described to be associated with the uptake of drugs, xenobiotics and compounds associated with endogenous metabolism (Hagenbuch and Gui, 2008; Obaidat et al., 2012; Stieger and Hagenbuch, 2014). Notably, ubiquitously expressed *SLCO2A1, SLCO2B1, SLCO3A1*, and *SLCO4A1* genes have been identified in ovarian cancer (Tamai et al., 2000). In addition, we previously detected SLCOs with a more restricted localization in normal tissue using samples from different ovarian cancer subtypes. These included the following: (i) *SLCO1B1* and liver-type *SLCO1B*3 (lt-*SLCO1B3*), which are normally expressed in the liver; (ii) *SLCO4C1*, which is highly expressed in the kidney; (iii) testis-specific *SLCO6A1*; and (iv) *SLCO5A1*, which is more widely distributed in the body (Svoboda et al., 2011). Novel *SLCO1B3* variants were identified in cancerous tissues. A cancer-type variant with a distinct 5′region, termed cancer-type (ct)-*SLCO1B3* coding for the cancer-type (ct)-OATP1B3 transporter was identified as the main OATP1B3 variant in colon, lung (Sun et al., 2014) pancreatic (Thakkar et al., 2013), and also ovarian cancer (Alam et al., 2018).

Thus far, various members of the OATP family (OATP1A2, OATP1B1, lt-OATP1B3, OATP2B1, OATP3A1, OATP4A1, and OATP5A1) have been identified as transporters for the 17β-estradiol (E2) precursor, estrone sulfate (E1S) and/or dehydroepiandrosterone sulfate (DHEA-S). Subsequently, OATP-mediated uptake of precursors may influence the progression of hormone-sensitive cancer subtypes. Of note, expression of OATP2B1 and OATP1A2, two transporters for steroid hormones, has been identified as important for the progression of hormone-dependent breast cancer (Banerjee et al., 2012; Matsumoto et al., 2015) and may also be significant for HGSOC. As serous ovarian carcinomas typically arise in postmenopausal women following the termination of ovarian estrogen synthesis, expression of OATPs might be important for providing E2 to hormone-dependent tumor cells. For an optimal estrogen turnover, uptake transporters must collaborate with the ABC-efflux transporter for the excretion of estrogen metabolites (Buxhofer-Ausch et al., 2013; Mueller et al., 2015; Rižner et al., 2017). After inactivation of E2 and other estrogens, for which conjugation through to sulfate via estrogen-preferring sulfotransferase is an important step, the conjugates are excreted via ATP-dependent drug efflux pumps (ABC-transporters), such as ABCC2 and ABCC3. Therefore, the expression of *SLCOs*/OATPs and ABC-efflux pumps could be of relevance in the progression of estrogen-sensitive HGSOC (Rižner et al., 2017).

OATPs may also be considered important for the accumulation of cytotoxic drugs in tumor cells; however, ABC-efflux pumps can limit the exposure of cells to their toxic effects. This suggests an influence of transporter gene expression on the efficacy of chemotherapeutic drugs (Ween et al., 2015; Bugde et al., 2017). From all established members of the OATP family, lt-OATP1B3 has been extensively characterized for its function as an uptake transporter for several established OATP substrates and for its role in paclitaxel uptake (van de Steeg et al., 2013). Because the OATP variant has poor transport capacity compared to the lt-OATP1B3, paclitaxel uptake by ct-OATP1B3 into ovarian cancer cells is unlikely (Thakkar et al., 2013; Sun et al., 2014; Furihata et al., 2015; Sissung et al., 2017; Alam et al., 2018).

ABCB1 (P-glycoprotein), which is encoded by the *ABCB1* gene, has been found to be significant in the efflux of taxanes from cells (Szakács et al., 2006). Notably, a previous study revealed that, although reduced *ABCB1* expression levels in epithelial ovarian cancer were related to longer progression-free survival of patients (Elsnerova et al., 2016), the role of P-glycoprotein in epithelial ovarian cancer is uncertain. Similarly, the expression of a number of ABC-transporters has been related to drug resistance and has been indicated to limit the exposure of cells to cytotoxic drugs. Furthermore, following platinum- and taxane-based standard chemotherapy, the upregulation of *ABCC2, ABCC3, ABCC4*, and *ABCC10* genes, which code for their respective ABC-transporters (multidrug resistance-related proteins MRP2, 3, 4, and 10, respectively) can contribute to drug resistance (Auner et al., 2010). Previous findings have suggested that the expression of a number of genes in the OATP and ABC-transporter families can be induced by the pregnane X receptor (PXR), which can be activated by many drugs and chemicals. Therefore, a possible co-expression of PXR and transporters may influence drug resistance. Furthermore, PXR expression has been related to ovarian cancer progression in previous studies (Nymoen et al., 2015; Dong et al., 2017).

Another possible important factor regarding OATPs and ABC-efflux pumps in ovarian cancer is their role in the regulation of the pro-inflammatory prostaglandin PGE_2_. After synthesis via the cytosolic PG-synthase 2 (COX2), PGE_2_ is exported by ABCC4 to the extracellular space for plasma membrane receptor binding. For the degradation of PGE_2_ via the prostaglandin-dehydrogenase (PGDH), a re-uptake into cells by PG transporters, including OATP2A1, is required. The collaboration between OATP and ABC-transporters together with prostaglandin-synthesizing/degrading enzymes has been proposed previously (Nomura et al., 2004; Holla et al., 2008; Kochel and Fulton, 2015).

Based on the proposed roles of individual OATPs to modify estrogen homeostasis, confer resistance to anticancer drugs and control the levels of pro-inflammatory prostaglandins, we sought to identify networks of directly correlated genes; *SLCOs* with genes coding for selected ABC-transporters, PXR, associated enzymes and the markers for HGSOC subtypes ERα/ERβ/HER-2. Providing further data on the expression of *SLCOs* and related genes to the well-established clinicopathologic prognostic parameters may allow improved risk stratification for patients with HGSOC because their specific expression pattern may reflect the heterologous nature of these tumors. The specific expression pattern may also point to the origin of the tumors and the pattern of peritoneal metastasis, which may influence the prognosis of patients. Therefore, the mRNA expression levels of SLCOs and the putative functionally-related genes were assessed in the present study using RT-qPCR in a cohort of 135 patients with late-stage HGSOC and compared with 21 benign ovarian cysts.

## Materials and methods

### Patients

Tumor samples of epithelial ovarian cancer (EOC) were collected in the course of the European Commission's 6th framework program project OVCAD (Ovarian Cancer: Diagnosis of a silent killer; grant agreement no. 018698). Contributors were: Department of Gynecology at Charité, Medical University Berlin, Germany; The Department of Obstetrics and Gynecology and Gynecologic Oncology, University Hospital Leuven, Belgium; Department of Gynecology and Obstetrics, University Medical Center Hamburg-Eppendorf, Hamburg, Germany; and The Department of Obstetrics and Gynecology, Medical University of Vienna, Austria. Benign ovarian cysts were collected at the Department of Obstetrics and Gynecology, Medical University of Vienna, Austria.

The study protocol was approved by the Ethics Committees of the participating institutions (approval nos. EK207/2003, Berlin; ML2524, Leuven; HEK190504, Hamburg; EK366/2003 and EK260/2003, Vienna). Permission to characterize new molecular prognostic factors for patients with advanced EOC was given by the Ethics committees and informed consent was obtained from all patients.

Information on clinic-pathological characteristics was documented by experienced clinicians and pathologists at the respective centers and the data are summarized in Table 1 (see Chekerov et al., 2013). Only patients with advanced stage (FIGO stage III-IV) and high grade (>1) serous tumors were included in the present study. Patients with additional malignancies were excluded. For comparison, samples from benign ovarian cysts were taken from patients (median age 53 years; interquartile range 39.5–65.5 years). All patients with EOC received standard first-line chemotherapy with platinum- and taxane-based chemotherapeutics after debulking surgery. Optimal cytoreduction with absence of residual disease was defined as macroscopically complete resection of tumor material. This was achieved in 71% of cases. The median age of cancer patients at diagnosis was 56 years (interquartile range, 49–67 years). The median follow-up time was 72 months with a median survival time of 48 (25.5–65.0) months. There were 92 cases of death (61%, all related to EOC) within the follow-up period.

**Table 1:** Clinicopathological characteristics of patients with high-grade serous epithelial ovarian cancer (HGSOC) and benign ovarian cysts.

**HGSOC Patients**	**135 (100%)**
**Age at diagnosis [years]**	
Median (IQR)	56 (49–67)
Overall Survival [months]	
Median (IQR) Number of deaths (%)	48.0 (25.5–65.0) 82 (61%)
**FIGO STAGE (%)**	
III	109 (81%)
IV	26 (19%)
**HISTOLOGICAL GRADING (%)**	
G2	32 (24%)
G3	103 (76%)
**RESIDUAL TUMOR AFTER**	
DEBULKING SURGERY (%)^*^	
No	94 (71%)
Yes	39 (28%)
**Benign Ovarian Cysts Patients**	**21**
**Age at diagnosis [years]**	
Median (IQR)	53 (39.5–65.5)

* *Data from 2 patients are missing; IQR, interquartile range*.

### RNA isolation and reverse transcription-quantitative polymerase chain reaction (RT-qPCR)

Tumor tissue was immediately frozen in liquid nitrogen and stored frozen until further use. For RNA isolation, approximately 100 mg of tissue was used as described previously (Svoboda et al., 2016). RNA was purified using the RNeasy Minikit (Qiagen Gmbh, Hilden, Germany) and quantified with a bioPhotometer (Eppendorf AG, Hamburg, Germany) and quality was assessed on an RNA Nano chip (Agilent Technologies Inc., Santa Clara, USA) and an Agilent 2100 Bioanalyzer.

Reverse transcription of 0.5 μg total RNA was performed using the High Capacity cDNA RT kit (ABI, Thermo Fisher Scientific, Inc., Waltham, MA, USA). To account for various cell types within the ovarian cancer tissues, out of a panel of 12 housekeeping genes (geNorm kit; Primer-Design Ltd., Southampton, UK) the following genes *ACTB, TOP1, UBC*, and *YWHAZ* were identified as the most stably expressed genes and were therefore were appropriate reference genes. Primers for genes of interest were purchased from ABI (Supplementary Table 1). The assay kit for ACTB was from ABI; assays for the reference house-keeping genes *TOP1, UBC*, and YWHAZ were purchased from PrimerDesign Ltd.

RT-qPCR analysis was performed on ABI 7900HT instrument equipped with SDS 2.3 software (Applied Biosystems) as described (Svoboda et al., 2016). Human Universal Reference Total RNA (Clontech Laboratories) was used for calibration.

Results were calculated according to the ΔΔCq method using the DataAssist™ v2.0 Software (ABI). Relative quantities (RQ) were normalized to the geometric mean of the four reference genes *ACTB, TOP1, UBC*, and *YWHAZ*. Values were shown relative to the mean value calculated from the calibrator samples. Samples with quantification cycle values (Cq) >35 were considered as not expressed (undetectable).

### Immunohistochemical staining of OATP5A1 on HGSOC sections

Tissue sections (4 μm) from tumors from HGSOC patients were analyzed. Samples were collected between 2006 and 2008. For immunohistochemical staining, the polyclonal rabbit anti-OATP5A1 antibody against OATP5A1 (HPA05062, Atlas antibodies, Stockholm, SW) was used in a dilution of 1:25. After deparaffinization with the Epitope Retrieval Solution DEPP-9 pH 9 (Eubio, Vienna, AT), samples were rehydrated, and treated with 3% H_2_O_2_. A blocking solution (Ultra V Block; TA-015HP, Eubio) was applied for 7 min and then the primary anti-OATP5A1 antibody was added for 1 h at room temperature. A rabbit immunoglobulin was applied as negative control. For enhancement, slides were incubated with primary antibody enhancer (Primary Antibody Enhancer; TL-015-PB, Eubio) for 10 min followed by a HRP Polymer (HRP Polymer; TL-015-PH, Eubio) for 15 min. Subsequently, slides were incubated with diaminobenzidine, which acted as a chromogen, and counterstained with hematoxylin before mounting.

Immunofluorescence staining was performed as described previously (Wlcek et al., 2011). OATP5A1 antibody HPA05062 (Atlas Antibodies) was applied in the double-immunofluorescence staining experiments at a dilution of 1:50 with a monoclonal antibody against cytokeratin 19 (AP06201PU-N, Origene, Rockville, MD) at a dilution of 1:500. Anti-mouse Alexa 568 and anti-rabbit Alexa 488 antibodies (Invitrogen, Thermo Fisher Scientific, Waltham, MA) were applied. Nuclei were counterstained with DAPI.

### Statistics

**Figure 1:**
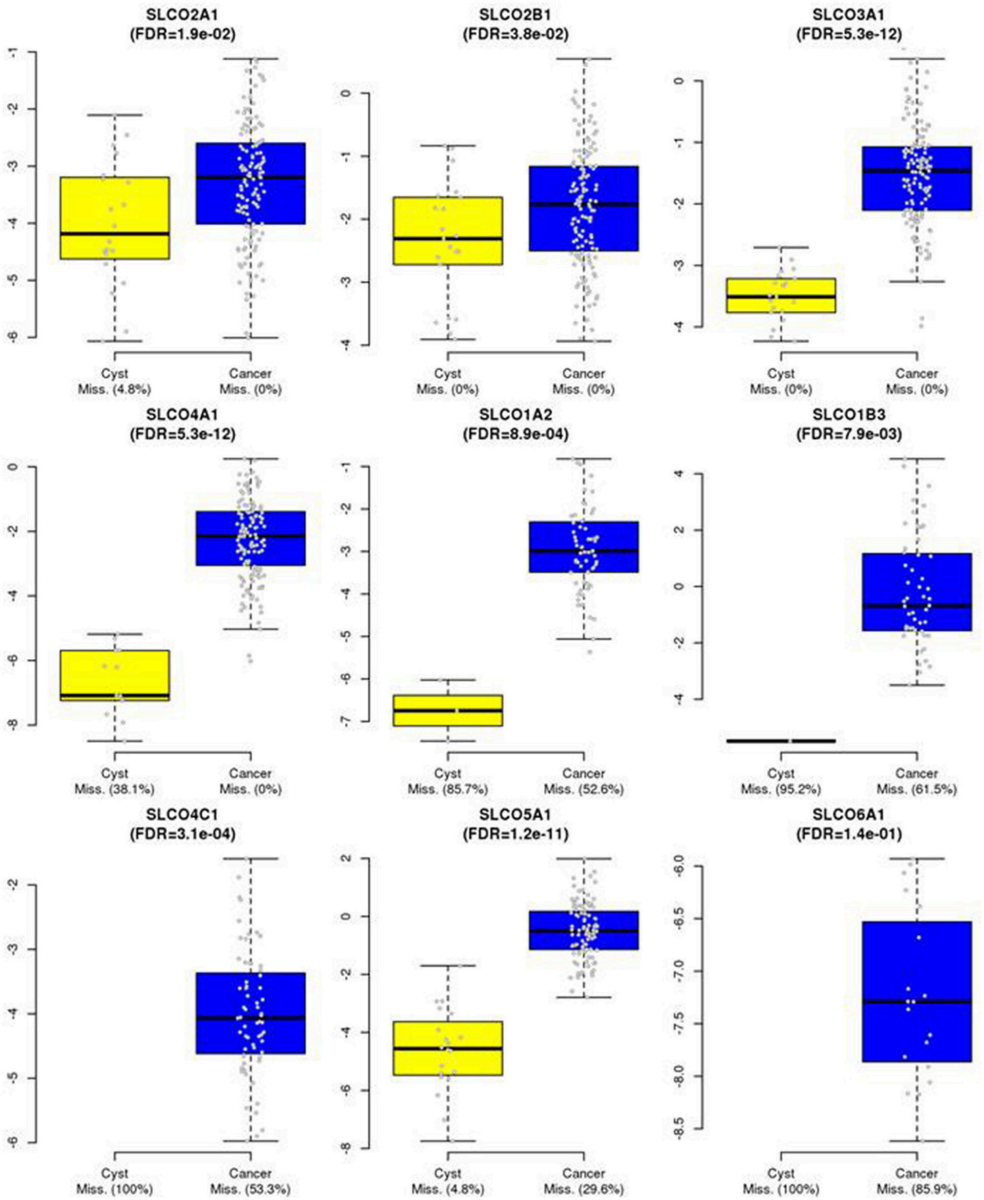
Expression of *SLCOs* in HGSOC tissues and benign ovarian cysts. *SLCOs*, which were expressed in a significant number of the 135 HGSOC samples were also assessed by RT-qPCR in 21 tissues from benign ovarian cysts. Boxplots are shown and missing rates in cancer samples and benign cysts are indicated in the x-axis legends. For certain genes in some samples, gene expression was below detection limit. Therefore, the boxplots represent only detectable values of the corresponding expression levels in the groups (*cf*. Materials and Methods). False Discovery Rates (FDRs) of *p*-values were calculated using a *two-part* Wilcoxon test (combining a test for continuous values with a test comparing point-mass proportions). Corrections for multiple testing were done by the Benjamini-Hochberg procedure.

Normalized gene expression values were used as log_2_ transformed ΔΔCq values and represented with two variables if undetectable samples (Cq values >35) were present: One variable containing the continuous log_2_ expression values (for all Cq <35) and one variable containing the information for detectable (= 1) or non-detectable (= 0) information in each sample (named with a suffix “_2K”) (*cf*. Supplementary Table 2). In all modeling approaches (Cox, logistic and linear regression) both variables were either included or excluded together. Samples with undetectable expression values were not included in the boxplots (Figure 1) but were always directing in the same direction (relatively more undetectable values in the group with the lower expression values, namely *SLCO2A1, SLCO4A1, SLCO1A2*, and *SLCO1B3*, but not for gene *SLCO5A1)*. Statistical testing was performed with a “two part” test combining a continuous component (Wilcoxon test) plus a point-mass (undetectable samples) (Taylor and Pollard, 2009) and corrected for multiple testing according to the Benjamini-Hochberg procedure (False Discovery Rates, FDRs).

For graphical Gaussian modeling (GGM) only gene expression values with no missing values were used with R-package GGMselect (0.1–12.1) (Giraud et al., 2012). GGM, also known as “covariance selection” or “concentration graph” modeling, is used to build gene association networks from expression data. The principle behind GGM is to use partial correlations as a measure of independence of any two genes which allows for distinguishing direct from indirect interactions. Only genes with complete expression values were included, to fulfill the requirements of GGM, which provided a Gaussian distribution of the data. The tuning parameter K for the penalty function was varied between 1 and 8 and the function selectFast [family = c(“C01,” “LA,” “EW”)] was used for optimizing the model employing the C01 algorithm, the Lasso-And (LA), and the Adaptive lasso (EW) families (https://cran.r-project.org/web/packages/GGMselect/vignettes/Notice.pdf). At each K the corresponding gene-networks were summarized by the first principal components and used together with all remaining single gene expression values that were not summarized in a network and the clinicopathological parameters. The LASSO Cox regression models were determined by grouped LASSO regression with R-package grpregOverlap 2.2-0 (Zeng and Breheny, 2016) at each K, optimizing lambda by minimization of the negative log likelihood (α = 1). The percentages of explained variation (PEV) of the Cox-regression models at each K-value were calculated by using the R-package Surev (v1.2) (Dunkler et al., 2007; Lusa et al., 2007) and maximized over K (*cf*. Supplementary Figure 1). PEVs given in Table 2 were also calculated using this R-package. Hazard ratios, confidence intervals, and *p*-values of all predictive factors (from simple or multivariable models) were summarized in forest plots (R-package, forestplot 1.8). In cases of simple models where two variables have to be considered (continuous and _2K), the *p*-values of the Wald test with two degrees of freedom were shown for both variables.

**Table 2:** Percentages of explained variation (PEV) of factors of/ and the final LASSO Cox regression model.

**Clinic/Genes**	**PEV**	**Partial PEV**	**Total PEV**
Age	12.80%		
FIGO stage			
Residual tumor			
*SLCO4C1* (categorical and continuous)		0.99%	
*SLCO5A1* (categorical and continuous)		5.08%	
*SLCO1B7*		0.32%	
*SLCO2A1*		1.12%	
*ABCC2*		0.84%	
*ESR1*		1.08%	
*ABCB2/ABCB3/ABCC4/HER2*		0.08%	
			23.13%

**Figure 2:**
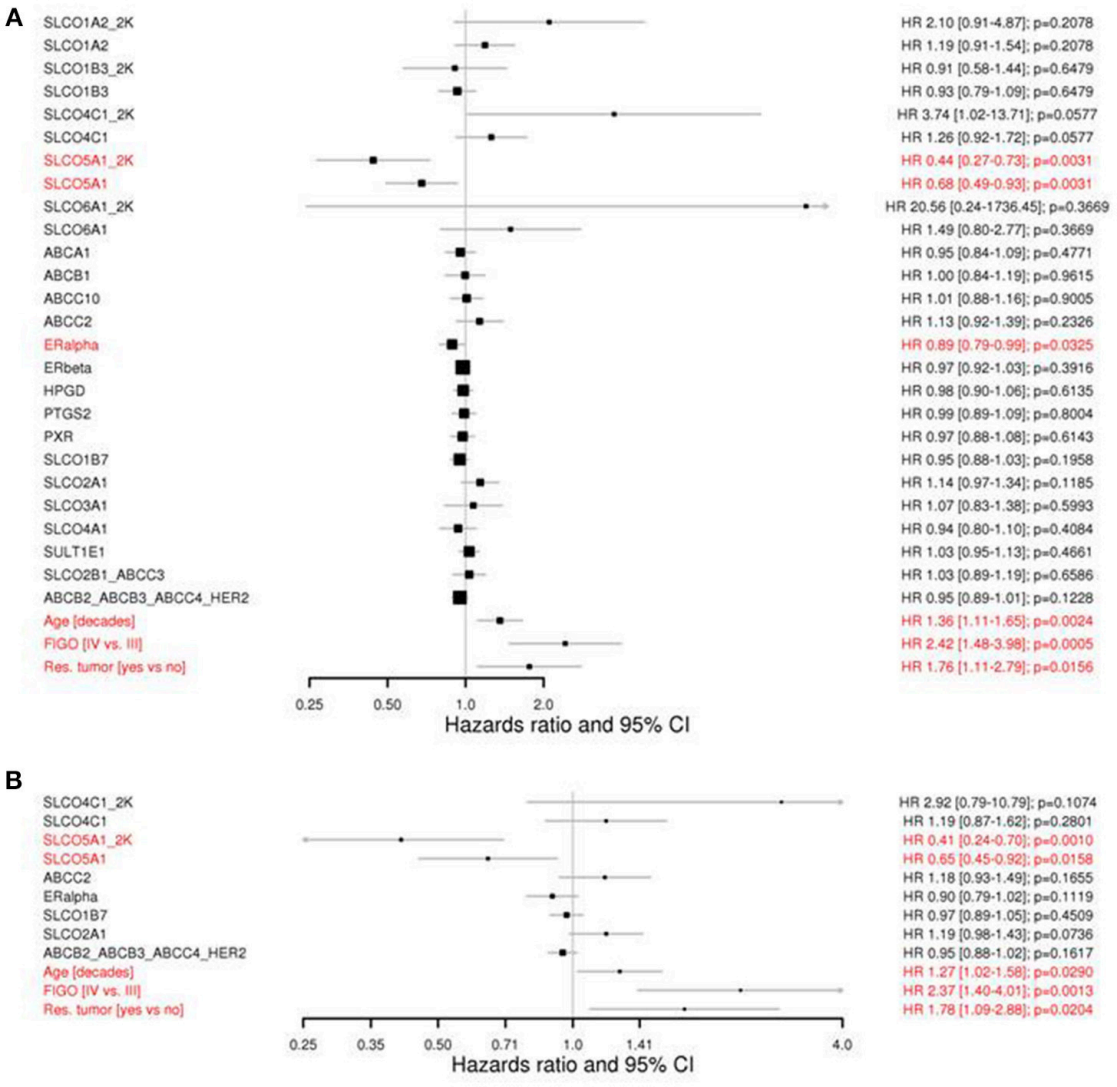
Forest plots showing the univariate Cox regression analyses **(A)** and the LASSO COX regression model for overall survival **(B)**. Current clinicopathological factors in ovarian cancer, including age, FIGO stage and residual tumor after debulking surgery are shown. Genes in networks are indicated by “_” between their names. “_2K” demonstrates the dichotomized variable indicating either expressed or undetectable (*cf*. Methods). *P*-values were from the 2 degrees of freedom Wald test if two variables, a continuous and a “_2K” were considered together. HR, hazard ratio; CI, confidence intervals; FIGO, International Federation of Gynecology Obstetrics; OS, overall survival. Note that *SLCO5A1* expression is significantly associated with improved prognosis in both analyses.

*The corresponding PEVs for the final multi-variable Cox model are shown (starting from 12.80% for the model including only the three clinicopathological factors). After including all seven additional predictors, a PEV of 23.13% was reached. The categorical and continuous variables for SLCO4C1 and SLCO5A1 were used together (see Figure 2)*.

To estimate the association of the single gene expression levels and gene-network principal components (calculated from the GGM selected networks) with molecular characteristics known to be important in HGSC biology, which included a molecular subclassification system (Yoshihara, “Yosh”) (Yoshihara et al., 2009), a signature indicating tumor spread types, non-miliary and miliary (“nM_M”; Auer et al., 2015), a signature indicating putative origin of HGSOC, either fallopian tubes or ovaries (“O_T”; Auer et al., 2017), and a signature indicating the epithelial-mesenchymal status (“EMT”; Miow et al., 2015), grouped LASSO logistic (Yoshihara subclasses 1 and 2) and linear (all other signatures) regression analyses were employed. Simple Spearman's rank correlation coefficients are shown in Table 3. The overlap with Yoshihara subclasses and the gene signatures were calculated from the corresponding microarray expression values that were previously determined in our lab (Pils et al., 2012). Overlapping genes related to the overall survival (“OS”) and the four subclassification/gene signatures were illustrated by a Venn diagram (R-package VennDiagram, v1.6.19). All calculations were performed in R version 3.4.3 (R Core Team, 2018).

**Table 3:** Spearman rank correlation coefficients.

**Gene/Network/Clinic**	**nM_M**	**O_T**	**EMT**	**Yoshihara**
*ABCB2/ABCB3/ABCC4/HER2*	−0.01	0.02	0.08	−0.19
*ABCC2*	0.02	−0.01	−0.01	0.06
*ESR1*	−0.01	0.12	−0.12	−0.15
*SLCO1B7*	0.01	0.06	0.03	0.05
*SLCO2A1*	−0.16	0.02	−0.09	0.28
*SLCO4C1*	−0.08	0.12	0.04	−0.13
*SLCO5A1*	−0.09	−0.14	0.00	−0.13
Age	−0.13	0.04	0.01	0.12
FIGO	−0.01	−0.09	−0.17	0.04
Residual tumor	−0.06	0.02	0.04	0.12
*ABCA1*	−0.07	0.04	−0.13	0.21
***ABCB1***	−0.10	0.10	0.04	0.00
***ABCC10***	0.02	0.08	0.01	0.04
*ESR2*	−0.04	−0.05	−0.15	−0.26
*HPGD*	−0.18	0.08	−0.07	0.13
***PTGS2***	0.01	0.00	−0.12	0.05
*PXR*	−0.11	0.05	−0.02	−0.21
*SLCO1A2*	−0.22	−0.07	−0.10	−0.04
***SLCO1B3***	0.03	0.03	0.14	0.02
*SLCO2B1/ABCC3*	−0.05	0.11	−0.04	0.28
*SLCO3A1*	−0.10	0.13	−0.04	−0.08
*SLCO4A1*	0.07	0.10	0.19	−0.08
***SLCO6A1***	0.02	−0.08	−0.01	−0.03
*SULT1E1*	−0.06	0.11	−0.06	0.02

*Data are given for (i) gene expressions, the first principal components of the co-expression networks, and clinicopathological parameters with (ii) gene signatures indicating molecular characteristics: peritoneal spread types, non-miliary or miliary (nM_M), positive value: high expression with M spreading; putative tumor origin, ovarian or fallopian tube (O_T), positive value: high expression with T origin; the epithelial-mesenchymal status (EMT), positive value: high expression with a more epithelial characteristic; and the Yoshihara (Yosh) molecular sub-classification system, high expression with subclass 2. Gray labeled are those genes, which were selected by the grouped LASSO approach predicting corresponding molecular characteristic. Bold printed are all genes/networks not present in any predictive LASSO regression model*.

## Results

### Analysis outline

The expression levels of genes coding for organic anion transporting polypeptides OATPs (*SLCOs, n* = 12), ABC-efflux pumps related to drug resistance (*n* = 8), the nuclear receptor PXR and three enzymes with possible relevance to the putative function of the transporters in the turnover of prostaglandins and estrogens (*PTGS2, PGDH*, and *SULT1E1)* were studied in samples from serous ovarian cancer together with *ESR1* coding for ERα, *ESR2* coding for ERβ and *HER-2* (Supplementary Tables 1, 2). Notably, the chosen enzymes PTGS2 and PGDH were used because of the importance of PTGS2 and PGDH in regulating PGE2 levels and their potential influence on inflammatory processes in cancer (Nomura et al., 2004), whereas SULT1E1 was used because it was shown that higher SULT1E1 protein levels were associated with an improved OS rate in HGSOC (Mungenast et al., 2017).

The main aim of the analysis was to elucidate the independent impact of the expression of these genes on OS with regard being given to the typical clinicopathological parameters known to predict outcome: age, FIGO stage IV vs. III, and residual tumor mass after debulking surgery (yes vs. no). Genes, which were expressed below the detection limit in some samples were represented by two variables in all models [one categorical variable (“_2K”) indicating expressed or not expressed (i.e., below detection limit) and another numerical variable for those samples where expression values were available, and both variables were forced to be included into the model either together or not at all]. To narrow down the number of predictors and to obtain biological information regarding the putative regulatory networks, we tried to use a method used to distinguish direct from indirect interactions by constructing the GGMs, from the expression data, only including genes with complete expression values (no samples below the detection limit). For GGM building, the tuning parameter K was varied between meaningful borders (Pils et al., 2012; Auer et al., 2015, 2017; Kurman and Shih Ie, 2016; Karam et al., 2017; Labidi-Galy et al., 2017; Salomon-Perzynski et al., 2017; Sukhbaatar et al., 2017) and selected according the maximized proportion of explained variation (PEV) of the corresponding LASSO Cox regression models for OS. The networks of directly correlated genes, as determined by the GGM procedure at each tuning variable value, were represented by the first principal component (PC1) of the network-gene expression values during model building (Supplementary Figures 1, 2).

To determine the impact of the genes or co-regulated networks (GGM networks) on the different characteristics of HGSOC tumors [related to a putative origin in T or O, molecular subclass, peritoneal tumor metastasis characteristic (nM or M) and EMT status], we used grouped LASSO logistic or linear regression modeling.

### mRNA expression in HGSOC (RT-qPCR)

Reverse transcriptase RT-qPCR was performed on total RNAs isolated from the tumor samples from 135 patients with advanced (FIGO stage III/IV) HGSOC (Supplementary Table 1). Expression levels were calculated from the log2 expression values of the respective genes normalized to the geometric mean of four stably expressed genes (*ACTB, TOP1, UBC*, and *YWHAZ*) for individual samples. Data for all genes are summarized in Supplementary Table 2. As expected from previous findings on *SLCO* expression in various ovarian cancer subtypes (Svoboda et al., 2011), *SLCO2A1, SLCO2B1, SLCO3A1*, and *SLCO4A1* were detected in all samples, while expression of the other 8 *SLCOs* was undetectable (requiring >35 cycles in the real-time RT-PCR) in a varying number of samples within the collective. Four *SLCOs*, namely *SLCO1A2, SLCO1B3, SLCO4C1*, and *SLCO5A1* were expressed (detectable) in a larger number of samples (47.4, 38.5, 46.7 and 70.3% of samples, respectively), but no discrimination was made for *SLCO1B3* variants (*lt-SLCO1B3* and *ct-SLCO1B3*). Three *SLCOs* that demonstrated a unique expression pattern in healthy tissues (liver, testis and brain) were also detected in a small number of ovarian cancer samples: “liver-specific” *SLCO1B1* in 1.5%, “testis-specific” *SLCO6A1* in 14.1% and *SLCO1C1*, which codes for the “brain thyroid transporter,” in 3% of samples.

In all samples of our cohort we detected the expression of *SLCO1B7*, which was previously considered a pseudogene but has recently been characterized for protein coding (Malagnino et al., 2018). This was a notable finding as the expression of *SLCO1B7* in normal tissues is considered to be high only in liver (https://www.ncbi.nlm.nih.gov/gene/338821).

mRNA expression levels of other genes investigated (the ABC-transporters coding *ABCA1, ABCB1, ABCB2, ABCB3, ABCC1, ABCC2, ABCC3, ABCC4*, and *ABCC10*, as well as *ESR1, ESR2, HER-2, PXR, PTGS2, PGDH*, and *SULT1E1)* were detectable in all samples (Supplementary Table 2).

### Comparison of *SLCO* expression between HGSOCs and benign ovarian cysts

By comparing the expression of *SLCOs* in HGSOC with that in benign ovarian cyst samples, we found that the expression values of *SLCOs* were generally higher in HGSOCs compared with benign tumors. Excluded from the calculation were *SLCO1B1* and *SLCO1C1*, which were rarely expressed in HGSOC tumor tissues (Supplementary Table 2) and were never observed in benign cysts (data not shown). All other data were used for estimating the differences between benign cysts and tumor tissues were presented as boxplots (Figure 1). Corresponding significance values (FDRs; Mann-Whitney-Wilcoxon tests corrected for multiple testing) and missing rates in benign cysts and cancer samples were shown. The data indicated that all analyzed *SLCO* genes exhibited significantly increased expression levels (and/or were more often detectable) in cancer compared with benign tissue samples (FDR ranging from 0.038 for *SLCO2B1* to 5.3 × 10^−12^ for SLCO3A1 and SLCO4A1, respectively). Importantly, *SLCO1B3* expression was only detected in 1/21 cysts (4.8%) and in 38.5% of HGSOC tissues (FDR = 0.0079). Although our analysis did not discriminate between lt-*SLCO1B3* and the cancer-type variant, it is most likely that ct-*SLCO1B3* might be the prominent isoform in the tumors investigated (Furihata et al., 2015; Thakkar et al., 2015; Alam et al., 2018). Both, *SLCO4C1* (FDR = 3.1x10^−4^) and *SLCO6A1* (FDR = 0.14) were not detectable in any of the benign ovarian cysts, however, there was no significant difference between the broad range of SLCO6A1 expression in the 14.1% of HGSOC samples compared with the benign ovarian cysts.

### Networks of directly correlated genes

We studied the impact of all genes investigated on the OS in the present HGSOC cohort. Clinicopathological parameters (age, FIGO stage IV vs. III, and residual tumor mass after debulking surgery) were always added as independent variables. Two *SLCO* genes were excluded from further analyses because they were only expressed in a limited number of samples (*SLCO1B1* and *SLCO1C1*). Five *SLCO* genes, which were expressed below the detection limit in several samples (Supplementary Table 2), were represented by two variables (one with suffix “_2K”) in all models (*SLCO1A2, SLCO1B3, SLCO4C1, SLCO5A1*, and *SLCO6A1*). To narrow down the number of predictors in the LASSO Cox regression models and to obtain biological information regarding putative regulatory connections, we constructed GGMs. As shown in the inset of Supplementary Figure 1, the tuning parameter K was varied between meaningful borders (Pils et al., 2012; Auer et al., 2015, 2017; Kurman and Shih Ie, 2016; Karam et al., 2017; Labidi-Galy et al., 2017; Salomon-Perzynski et al., 2017; Sukhbaatar et al., 2017) and the final used value determined by maximizing the PEV of the corresponding LASSO Cox regression model for OS was conducted for GGM building. The PEVs of the Cox regression models are shown for all used GGM tuning parameter values (Table 2). A steady increase of the PEV up to *K* = 4 was seen, with no further increase beyond 4. Therefore, the model at *K* = 4 was used for all further analyses, which yielded two putative co-regulated networks: *ABCB2/ABCB3/ABCC4/HER2* and *ABCC3/SLCO2B1* (Supplementary Figure 1). In Supplementary Figure 2, the correlation between genes within network 1 and network 2, respectively, are shown based on the model at *K* = 4.

### Influence of genes and networks on OS

In Figure 2 (**A**: simple, **B**: final multi-variable model), forest plots of Cox regression models for the 20 independent genes and the two networks at *K* = 4 for OS are presented. In addition to the three clinicopathological parameters age, FIGO stage and residual tumor with a statistically significant negative impact on OS, *SLCO5A1* was the only independent single gene with a significant positive impact on OS [HR, 0.44 (CI 0.27–0.73) and 0.68 (0.49–0.93) for _2K and the continuous variable, respectively; *p* = 0.0031; Wald test with two degrees of freedom]. A significant positive impact on OS was also observed for *ESR1* coding for ERα, which was as expected (Figure 2A; HR, 0.89; unadjusted *p* = 0.0325).

Notably, there was only *SLCO4C1* exhibited a trend for negative impact at _2K [HR, 3.74 (CI 1.02–13.71)] and at the corresponding continuous variable [HR, 1.26 (CI 0.92–1.72) *p* = 0.0577]. Similarly, a tendency for a negative impact was noted for *SLCO1A2* and *SLCO6A1*, and a positive impact on the OS for *SLCO1B3* and *SLCO1B7* was also indicated. However, because of the high variability of data and restricted expression values, no level of significance was reached. Furthermore, no significant independent influence on the OS in the univariate and multivariate analysis was demonstrated for the two identified gene networks *ABCB2/ABCB3/ABCC4/HER2*, and SLCO2B1/ABCC3 for the remaining genes.

In the multivariable analysis (Figure 2B), only *SLCO5A1* but not *ESR1* exhibited a significant positive impact on OS (HR, 0.41; *p* = 0.001 and HR, 0.65; *p* = 0.0158 for _2K and the continuous variable, respectively).

**Figure 3:**
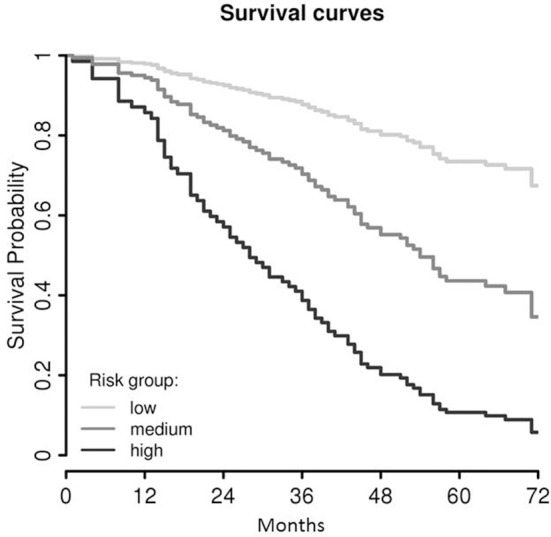
Survival curves of the final LASSO COX regression model (*cf*. Figure 2B), trichotomized according the terciles of the model value in low-, medium-, and high-risk groups. As these survival curves represent a multivariable Cox model, no censored patients are indicated.

Using the Cox regression model at *K* = 4, the probabilities of survival were estimated for patients categorized according to the terciles in subgroups, i.e., low-, medium- and high-risk (Figure 3). Five-year survival estimates were 73.5% [CI 63.5–85.0], 43.6% [CI 35.0–54.2] and 10.7% [CI 5.1–22.3], respectively.

Combining the results from both analyses (LASSO Cox regression and univariate Cox regression), we indicated that *SLCO5A1* has a significant prognostic effect on OS, with favorable outcomes associated with patients with high *SLCO5A1* expression.

The corresponding percentages of PEVs for the final multi-variable Cox model were shown, starting with 12.8% for the model, including the three clinicopathological factors alone (Table 2). It reached 23.13% after including all seven additional genes, which were found to have a prognostic impact (the categorical and continuous variables for *SLCO4C1* and *SLCO5A1* were used together, see Figure 2). Remarkably, *SLCO5A1* increased the PEV by 5.08%, while the contribution of *SLCO2A1* and *ESR1* was only 1.12 and 1.08%, respectively. However, these measures of added prognostic accuracy were determined on the same data set that was used for gene network and variable selection and could therefore be over-optimistic.

### Immunohistochemistry of OATP5A1 in ovarian cancer tissues

**Figure 4:**
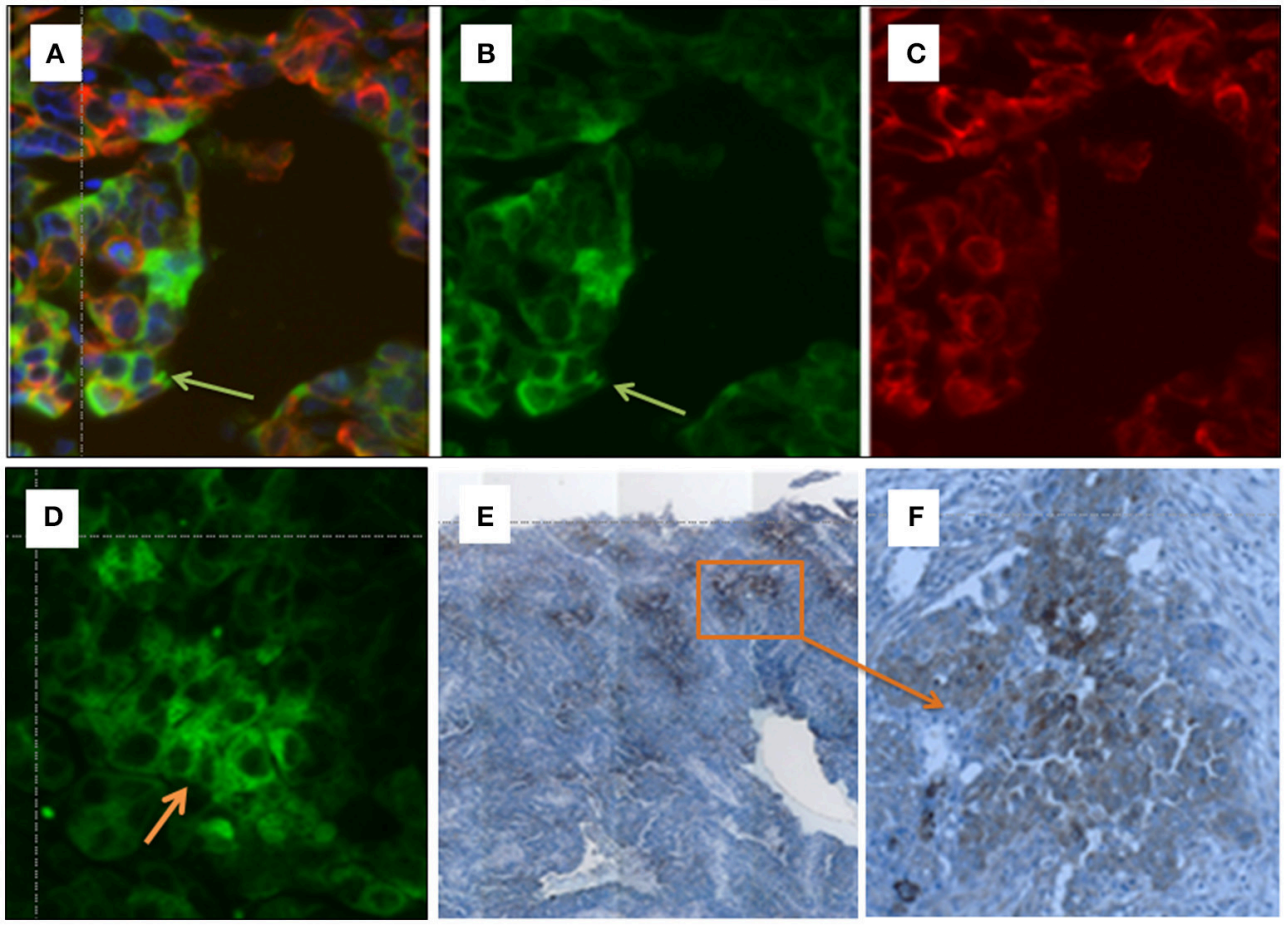
**(A–F)** Immunohistochemical detection of OATP5A1 in HGSOC tissue samples. Double immunofluorescence staining of OATP5A1 (green) and cytokeratin 19 (red) in paraffin-embedded sections from serous ovarian carcinomas **(A–D)**. **(A)** Merged image of OATP5A1 **(B)** and cytokeratin-19 **(C)**. The orange overlay color for co-localized OATP5A1 and cytokeratin-19 is only visible in some cells (yellow arrow) in the tumor. Note the staining of the cytoplasm and membrane with the antibody against OATP5A1 is visible in image **(B)**, and the predominantly cytoplasmic staining of OATP5A1 in image **(D)**. Immunohistochemical staining of OATP5A1 in HGSOC sections **(D,E)**. Overview on a HGSOC section (x10) revealing clusters of brown (OATP5A1)-stained cells in the tumor **(E)**. Clusters of OATP5A1-positive cells at a higher magnification, x40 **(F)**.

To validate the OATP5A1 protein expression in ovarian cancer tissues, the localization of the transporter in paraffin-embedded sections from serous ovarian cancer tissues was analyzed by using immunohistochemistry and immunofluorescence staining (Figures 4A–F). We used an antibody against OATP5A1, which was previously shown to detect the transporter by western blot analysis of OATP5A1 overexpressing Sf9 cells (Patik et al., 2015). Immunohistochemistry results indicated positive staining for OATP5A1 observed in a number of tumor cells clustered together in the tumor center, while other areas were negative for OATP5A1 (magnification 10x, Figure 4E). An area with OATP5A1-positive cells was also shown at a higher magnification (40x) (Figure 4F).

Double-immunofluorescence staining of green OATP5A1 (Figure 4B) and red cytokeratin-19 (Figure 4C), which was used as a marker for epithelial cells resulted in orange to yellow staining in some cells as a result from the overlay of green transporter with the red epithelial marker (Figures 4A–D). Predominately membranous staining or a more cytoplasmic staining pattern for OATP5A1 was visible in different specimens (Figure 4B vs. 4D). Interestingly, OATP5A1 was more frequently detectable in tumor cells with weaker cytokeratin-19 staining compared with that in cells with strong cytokeratin-19 staining (Figure 4A). This was also observed in other samples (*n* = 8) from HGSOC tumors (data not shown) revealing that OATP5A1 is not restricted to tumor cells with epithelial characteristics.

### Correlation with HGSOC subclassification systems and gene signatures

**Figure 5:**
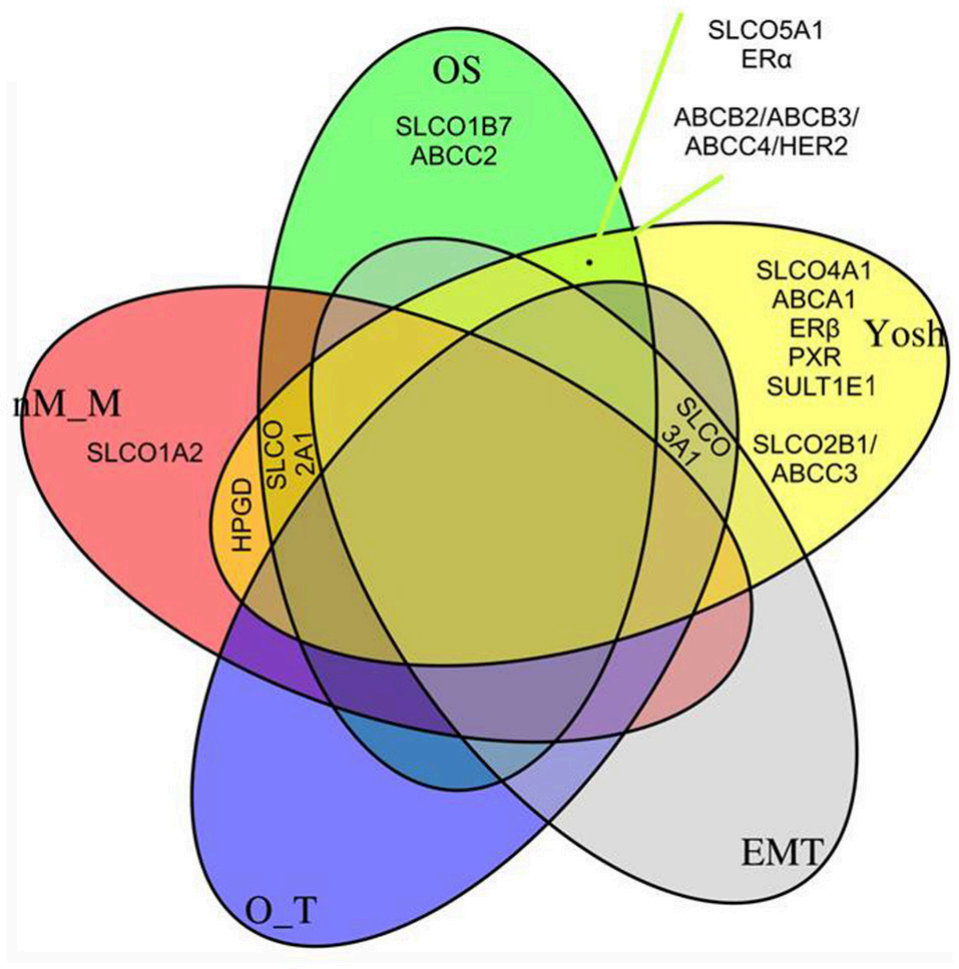
Quintuple Venn plot. Overlapping genes/networks from the LASSO Cox regression model (*OS*, Figure 2B), a logistic regression model indicating a molecular subclassification system published by Yoshihara et al. (2009), and linear regression models (a gene signature indicating the epithelial-mesenchymal transition status (*EMT*), a gene signature indicating the putative origin of the tumors (Auer et al., 2017; Sukhbaatar et al., 2017), either ovarian surface epithelium or fallopian tube secretory epithelial cells (*O_T*), and to a signature indicating either miliary (M) or non-miliary (nM) tumor spread (*nM_M*) (Auer et al., 2015, 2016, 2017; Bachmayr-Heyda et al., 2016; Sukhbaatar et al., 2017). See also Table 3, where simple Spearman's rank correlation coefficients of all combinations given in Figure are shown.

In the previous sections we have studied the impact of *SLCO*s, related genes and networks on OS. In this section we want to elucidate if the same genes and networks are related to published subclassification systems and gene signatures associated with specific characteristics of HGSOC (Table 3, Figure 5). Grouped LASSO logistic or linear regression modeling was used. The following characteristics were calculated from the gene expression data as described in the following references: A dichotomous molecular subclassification system published by Yoshihara et al. (Yoshihara et al., 2009) that was validated by us for OS (Auer et al., 2017); and three gene signatures correlating with (i) the EMT status (Miow et al., 2015), (ii) the putative origin of the tumors ovarian surface epithelium or fallopian tube secretory epithelial cells (O_T) (Auer et al., 2017; Sukhbaatar et al., 2017) and (iii) a signature indicating either military (M) or non-miliary (nM) tumor metastasis (nM_M) (Auer et al., 2015, 2016, 2017; Bachmayr-Heyda et al., 2016; Sukhbaatar et al., 2017). In Figure 5, the overlap of genes and networks used for the analysis (see also Supplementary Figure 2) with molecular characteristics of HGSOC (represented by these four gene signatures) was demonstrated in a Venn plot. Additionally, Spearman's rank correlation coefficients of all combinations are shown in Table 3.

Importantly, the positive impact of *SLCO5A1* on OS determined by the multivariate and univariate COX analyses correlated with its attribution to the Yoshihara subclass 1, which suggested a favorable OS. Consistent with our validation data (OS), within this group we identified *SLCO5A1, ESR1*, and the network *ABCB2/ABCB3/ABCC4/HER2*. Notably, *SLCO1B7 and ABCC2* corresponded with our own validated signature (OS).

Remarkably, *SLCO3A1* was associated with Yoshihara group 1 but also with a signature reflecting a putative origin from the ovaries rather than from the Fallopian tubes (O_T) and EMT. Notably, tumors to be considered of ovarian origin were also characterized with an improved prognosis. Since ovarian cancer cells express mesenchymal and epithelial markers simultaneously, transition from one state to another might be a continuous process in these cells (Auer et al., 2017), for which *SLCO3A1* might be a marker.

Interestingly, the genes coding for the prostaglandin transporter *SLCO2A1* and the PGE_2_-inactivating enzyme *HPGD*, respectively, overlap with the Yoshihara signature and the n-M signature for peritoneal metastasis. An overlap with the n-M_M signature was also observed for *SLCO1A2*. *SLCO2A1* additionally belonged to our OS signature. It was indicated that the N-M tumor metastasis pattern and a putative ovarian origin, rather than tubular origin, are associated with a more favorable prognosis (Auer et al., 2017), which would be in line with a role of *SLCO2A1* and *HPGD* implicated in the degradation of pro-inflammatory prostaglandins.

From the 21 genes investigated, 5 genes (*SLCO1B3, SLCO6A1, ABCB1, ABCC10*, and *PTGS2*) did not belong to any signature in the subclassification systems used in our LASSO regression models.

## Discussion

The present study investigated the impact of the expression of *SLCO* and related genes coding for ABC-transporters, PXR, ESR1/2, and HER-2 in serous ovarian cancer patients treated with debulking surgery and platinium- and taxane-based chemotherapy. The results indicated that all *SLCOs* were upregulated in HGSOC compared with benign ovarian cysts and it was identified that *SLCO5A1* may be a potential positive prognostic factor for OS of HGSOC patients. Furthermore, the present findings suggested two networks of *SLCO* and ABC-transporter coding genes (*SLCO2B1/ABCC3* and *ABCB2/ABCB3/ABCC3/HER-2*) and demonstrated the expression of *SLCO1B7* in ovarian cancer samples.

Higher expression of all *SLCOs* in samples from serous ovarian cancer compared with benign ovarian tumor samples suggests the importance of this *SLCO*/OATP family for cancer progression. Many studies have revealed that transporters of the OATP family are functionally expressed in cancerous tissues, where they may be critical for determining the local concentration of endogenous metabolites such as steroid hormones, mediators in the inflammatory pathway and others as well as drugs. Thereby they can influence cancer biology and progression.

Particularly expression *of SLCO5A1* is of interest, because by using a combination of LASSO Cox regression and univariate Cox regression models, we showed that higher *SLCO5A1* expression in tissues of HGSOC patients had a significant positive impact on overall survival, a thus seems to have protective functions. The significant association of high *SLCO5A1* expression with the Yoshihara subclass 1 is in agreement with the positive effects on OS from or study (Pils et al., 2012). Further support of our findings is coming from data from the Human Protein Atlas where a trend for a better survival probability for ovarian cancer patients is associated with higher OATP5A1 mRNA expression levels (https://www.proteinatlas.org/ENSG00000137571-SLCO5A1/pathology).

These data clearly suggest that further studies in a larger collective of HGSOC patients should be performed to further explore the prognostic value of *SLCO5A1* in HGSOC. Furthermore, there is a general lack of studies on a potential prognostic effect of OATP5A1 for cancer patients; however, *SLCO5A1* expression has been reported in a number of tumors and the localization of the OATP5A1 protein has been shown in liver and breast tumors (Kindla et al., 2011; Wlcek et al., 2011).

The data also raise the question whether OATP5A1 functions as a transporter for known OATP substrates into cells (Hagenbuch and Gui, 2008; Stieger and Hagenbuch, 2014). Notably, a recent study in *SLCO5A1* transfected Sf9 insect cells indeed showed that OATP5A1 is capable of mediating the uptake of E2-glucuronide at an acidic pH (Patik et al., 2015). Because extensive lactate secretion from the anaerobic glycolysis leads to an acidification of the extracellular tumor environment (Kato et al., 2013), uptake processes for potential estrogen precursors driven by the proton gradient might also be important for the progression of hormone-dependent ovarian cancer cells. It is worth to mentioning that pH-dependent transport of estrogen precursors was also described for the OATP2B1 in luminal A-like breast cancer (Matsumoto et al., 2015). Furthermore, the present results found that this OATP was upregulated in all HGSOC specimens investigated (see Figure 1).

The prominent cytosolic localization of OATP5A1 in ovarian cancer samples provides arguments against an important role of OATP5A1 in the transport of estrogen precursors or other substrates through the plasma membrane in HGSOC. For the uptake of compounds into the cells or extrusion of metabolites from cells to the extracellular space, plasma membrane localization would be required. However, OATP5A1 may still function as a transporter in intracellular organelles, which has been shown for a number of other transporters (Hediger et al., 2004). Also, intracellular sequestration of pharmacological agents may explain the increased resistance of HEK-293 cells to satraplatin after transfection with OATP5A1 (Olszewski-Hamilton et al., 2011). There is a strong possibility that OATP5A1 influences cancer progression by influencing cellular differentiation and migration directly. Studies in HeLa cells revealed that overexpression of OATP5A1 resulted in a reduction of cell proliferation and enhanced expression of genes that contribute to cell-cell adhesion, structural development and cell growth including desmocollin 3 and transglutaminase 2 (Sebastian et al., 2013).

However, a significant negative impact [a shift of HR to 3.74 (1.02–13.71; *p* = 0.047)] on the OS was demonstrated for *SLCO4C1* as a single gene in the present study. *SLCO4C1* encodes the “kidney-specific” OATP4C1, a transporter for the excretion of uremic toxins to the urine (Toyohara et al., 2009). Because excretion of these toxins is impaired through kidney damage, upregulation of this transporter may reflect the damage to endothelium and other structures. With disease progression, other organs may be subjected to destruction by these toxins. As result, increased levels of uremic toxins can also damage the endothelium in extra-renal tissues, which may lead to an upregulation of OATP4C1 in tumors. This may explain the expression of *SLCO4C1* in a number of HGSOC samples and its absence in benign ovarian cysts in the present findings.

It is unclear which mechanism(s) may lead to the upregulation of *SLOC* genes in serous ovarian cancer. On the molecular levels, many mechanisms for epigenomic alterations are known that can change the gene expression pattern (Furihata et al., 2015). Both, (gene specific) hypermethylation and (global) hypomethylation of the DNA are commonly observed in various types of cancers incuding ovarian cancer (Kwon and Shin, 2011). Previous studies examined the expression of ct-OATO1B3 mRNA and indicated that DNA hypomethylation caused upregulation and that hypermethylation resulted in the suppression of ct-OATP1B3 mRNA expression in cancer cell lines (Imai et al., 2013; Sun et al., 2014).

Different transcription factors have been identified in the regulation of OATP expression. Importantly, the mRNA expression of a cancer-specific OATP1B3 variant is induced by the hypoxia-inducible factor (HIF-1α), which mediates cell survival under hypoxic conditions (Han et al., 2013). Apart from the *SLCO1B3* gene, binding sites for HIF-1α were also found in *SLCO4A1*, while binding sites for different transcription factors including HNF-α, NF-AT, c-Myc, NF-κB were found in different *SLCOs* using *in silico* structural analyses (http://www.genecards.org). For OATP5A1 expression, (http://www.genecards.org/cgi-bin/carddisp.pl?gene=SLCO5A1) a p53 binding site in the promoter of *SLCO5A1* was identified *in silico*. Because *TP53* is mutated in >95% of HGSOCs, missense mutations in the DNA-binding domain of *TP53* might be related to lower levels of *SLCO5A1* in HGSOC samples, Whether there is a correlation between *TP53* mutations, *SLCO5A1* levels and the prognosis for HGSOC patients. Further studies are required to elucidate this.

In the present study we identified two networks between genes coding for OATP transporters and ABC-efflux pumps. Although only a marginal impact of both networks was indicated according to the HR in univariate and multivariate COX analyses in our study, the network *ABCB2/ABCB3*/*ABCC4/HER-2* was indicated to be associated with the Yoshihara group 1 and a more favorable prognosis (Yoshihara et al., 2009; Pils et al., 2012).

The first identified co-expression network revealed *SLCO2B1* was connected with the *ABCC3* gene coding for the ABCC3 protein (MRP3). Notably, ABCC3 is implicated in drug resistance but also mediates the efflux of many glucuronidated metabolites formed from drugs. Therefore ABCC3 may be important for estrogen inactivation. Interestingly, a joint activity for OATP2B1 with another ABC-efflux pump, ABCG2 (BCRP), was described in a study on the human placenta, where the two transporters were thought to mediate uptake and excretion of sulfated estrogen precursors (Grube et al., 2007). However, considering that OATP2B1 may be an uptake transporter for estrogens, it has been suggested that a number of OATPs (OATP1A2, OATP1B1, OATP1B3, OATP1C1, OATP3A1, OATP4A1, and OATP5A1) (Stieger and Hagenbuch, 2014) and also LST-3TM12 and OATP6A1 (Patik et al., 2015; Malagnino et al., 2018) are capable of transporting estrogen precursors. Furthermore, the substrate specificity for E1S and DHEA-S differs widely between individual members and cells investigated. In serous ovarian cancer, *SLCO1B1, SLCO1C1*, and *SLCO6A1* were observed in a low number of samples only. This suggests that the contribution of individual OATPs for providing estrogen precursors to cancer cells may depend on the specific expression pattern in a certain tumor entity and its local environment.

The second identified co-expression network in serous ovarian cancer exists between ABC-transporters *(ABCB2, ABCB3*, and *ABCC4)* and *HER-2*. In this network, *ABCB3* and *ABCB2* are coding for the functional transporters TAP1 and TAP2 as part of the immune system. Endogenous peptides are transported by the TAP-complex from the cytosol to the endoplasmic reticulum and loaded onto MHC I for antigen processing. A down-regulation of the HLA class I antigen presentation machinery (APM) decreases recruitment of tumor-infiltrating lymphocytes, which leads to a reduction in the antitumor activity of the immune system (Aust et al., 2017). We previously showed that programmed death-ligand 1 (*PD*-*L1*) also known as CD274 on tumor cells is associated with reduced MHC I (APM) expression in HGSOC. This indicates two mutually exclusive immune-evasion mechanisms in ovarian cancer: down-regulation of T-cell mediated immunity by PD-L1 expression and silencing of self-antigen presentation by down-regulation of the MHC I complex (Aust et al., 2017). The correlation of the expression of this network with Yoshihara subclass 1 with a better prognosis also corresponds to the correlation of low MHC I expression with bad prognosis. Higher levels of ABCB2 and ABCB3 in ovarian tumors were previously shown to be associated with longer survival of patients (Auner et al., 2010). Upregulation of *ABCB2* and *ABCB3* in the antigen presentation pathway may therefore improve antigen-specific active immunotherapy and the outcome of an antibody-based therapy against HER-2. The forth component in the network is *ABCC4*, which codes for the ATP-efflux-pump ABCC4 (MRP4). ABCC4 substrates include cyclic nucleotides (cGMP, cAMP), anticancer drugs such as camptothecins, methotrexate and prostaglandins. By mediating nucleotide transport, ABCC4 influences various purinergic signaling pathways, which depending on the local conditions, either inhibits or stimulates cell survival, proliferation, invasion and metastasis in various types of cancer (Choi et al., 2003) and may lead to an altered expression of *HER-2* (Chen et al., 2016). Furthermore, ABCC4 is an important efflux transporter for the PGE_2_ and may work with OATP2A1 and the PGE synthesizing/degrading enzymes *PTGS2*, and *PGDH* in the processing and degrading of PGE_2_ synthesized in the arachidonic pathway. However, no direct co-expression for *ABCC4* with these enzymes or *SLCO2A1* was observed in our study, which was in contrast to previous data from a colon cancer study (Nomura et al., 2004).

An additional important finding of the present study was that *SLCO1B7*, which is a poorly characterized member of the *SLCO* family 1 member, was present in all samples in the cohort. A recent *in silico* analysis revealed that trans-splicing of *SLCO1B7* and *SLCO1B3* produced a novel OATP family 1 member, LST-3TM12. Furthermore, heterologous expression of *SLCO1B3* and *SLCO1B7* in HeLa cells leads to LST-3TM12 protein expression, resulting in an increased cellular accumulation of E2-glucuronide and DHEA-S (Malagnino et al., 2018). However, we did not observe significant co-expression of *SLCO1B7* with *SLCO1B3* in our cohort, but co-expression with the cancer-specific variant might be possible (Furihata et al., 2015; Thakkar et al., 2015). Therefore, future studies are warranted to elucidate the expression pattern of *SLCO1B7* with ct-*SLCO1B3* in cancer to elucidate whether a functional LST-3TM12 protein in HGSOC exits and to gain information to its relation to drug resistance and cancer progression.

Taken together, we indicated an analysis of a small but well-documented number of patients to elucidate the OATP mRNA expression pattern in HGSOC. However, the approach used in the present study did not address the complete biological heterogeneity nor did it identify all related genes.

In conclusion, we indicated upregulation of various *SLCO* transporters in HGSOC compared with benign cysts. Furthermore, we identified a network of *SLCO2B1* and *ABCC3* with relevance for estrogen turnover and a further network consisting of genes encoding 3ABC-efflux pumps and HER-2 with relevance for immune regulation. Additionally, an overlap with previously described signatures for a more favorable prognosis of patients with HGSOC was demonstrated for *SLCO5A1*.

## Author contributions

TT, MS, RZ, and DC-T: Design and coordination of the study; MS, FM, DP, AG, and TT: Experimental part, analysis and interpretation of data; AG and DP: Bioinformatical and statistical analyses; TT, DP, MS, FM, with support of WJ, DM: Preparation of the manuscript; DC-T, RZ, IV, AV, JS, EB, and SM: Providing of sample and maintaining patient database. All authors read and approved the final manuscript.

### Conflict of interest statement

The authors declare that the research was conducted in the absence of any commercial or financial relationships that could be construed as a potential conflict of interest.

## Supplementary material

Supplementary Figure 1 Co-expressed gene networks. Genes used for GGM modeling are shown, which indicates the two networks that were revealed by the algorithm at the tuning parameter *K* = 4. The proportion of explained variation (PEV) values of the LASSO Cox regression models are shown for every tuning value between 1 and 8. As indicated, up to *K* = 4 a steady increase of the PEV was seen with no further increase beyond 4. Therefore, the model at *K* = 4 was used for all further analyses, which yielded two putative co-regulated networks: *SLCO2B1/ABCC3*, and *ABCB2(TAP1)/ABCB3(TAP2)/ABCC4/HER2*. These two networks were used for all analyses and represented by their first principal components (*cf*. Supplementary Figure 2).

Supplementary Figure 2 Scatter plots showing the correlations of genes of both co-expressed networks with their first principal components (PC). Size coded values in the top-right square represent correlation coefficients and significance levels are indicated by °*p* < 0.1, ^***^*p* < 0.001 (Spearman's correlations). Network 1: *SLCO2B1/ABCC3* **(A)**; *N*etwork 2: *ABCB2/ABCB3/ABCC4/HER-2* **(B)**.

Supplementary Table 1 List of TaqMan® Gene Expression Assays for RT-qPCR. Assays were purchased from Applied Biosystems (Thermo Fisher, Waltham, MA) for RT-qPCR.

Supplementary Table 2 Expression of *SLCOs* and genes coding for putative related ABC-transporters, enzymes, nuclear receptors, and HER-2 in 135 HGSOC samples.
