# Supplementary figures and images for: Clinical Significance of Organic Anion Transporting Polypeptide Gene Expression in High-Grade Serous Ovarian Cancer
Source: Front Pharmacol. 2018 Aug 7;9:842. doi: 10.3389/fphar.2018.00842 (PMC6090214; doi:10.3389/fphar.2018.00842)

Figure 1 (Supplement)

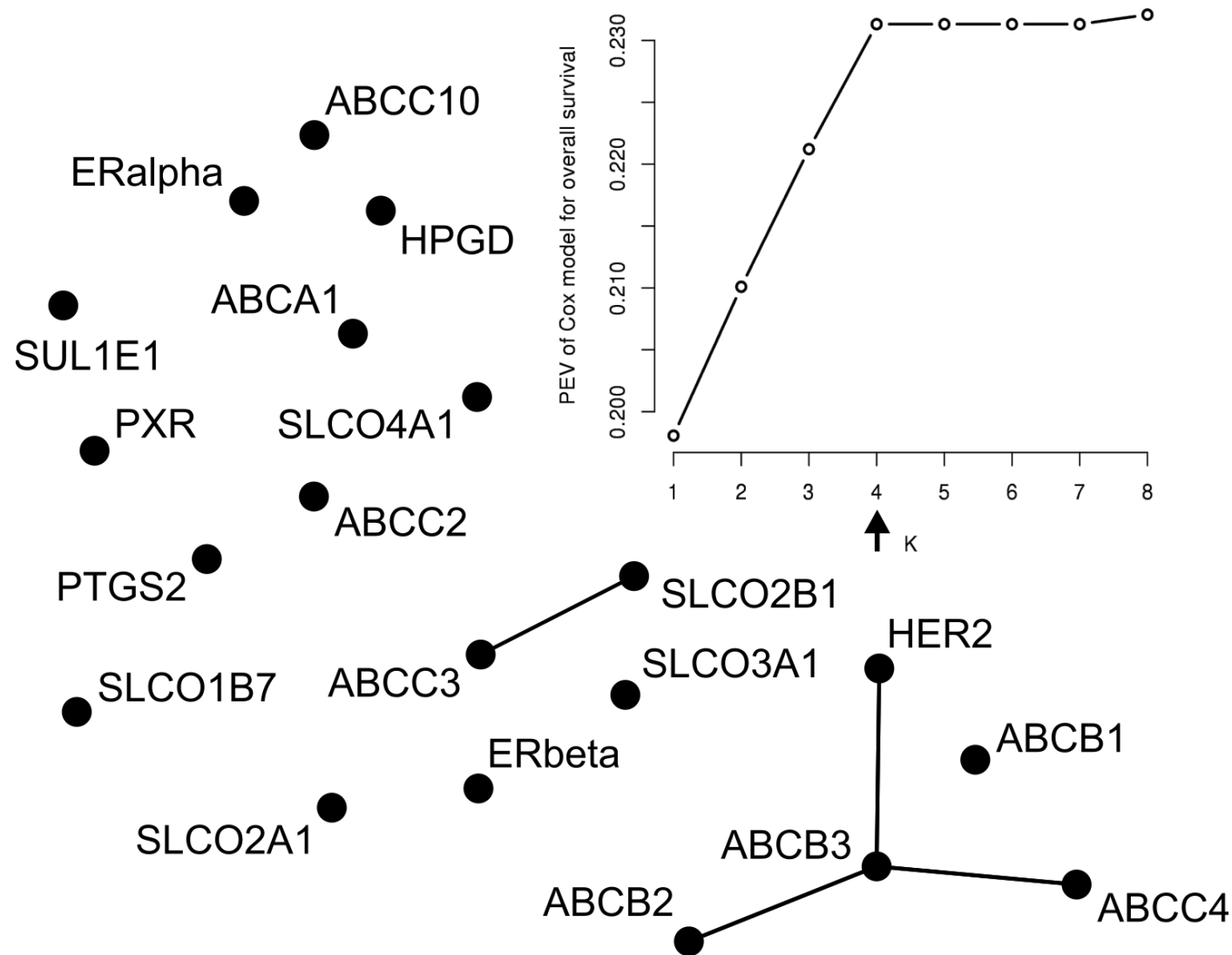

Supplement: Supplementary Figure 1 — Co-expressed gene networks. Genes used for GGM modeling are shown, which indicates the two networks that were revealed by the algorithm at the tuning parameter K = 4. The proportion of explained variation (PEV) values of the LASSO Cox regression models are shown for every tuning value between 1 and 8. As indicated, up to K = 4 a steady increase of the PEV was seen with no further increase beyond 4. Therefore, the model at K = 4 was used for all further analyses, which yielded two putative co-regulated networks: SLCO2B1/ABCC3, and ABCB2(TAP1)/ABCB3(TAP2)/ABCC4/HER2. These two networks were used for all analyses and represented by their first principal components (cf. Supplementary Figure 2). [file Image_1.PDF]

Figure 2  
(Supplement)

A

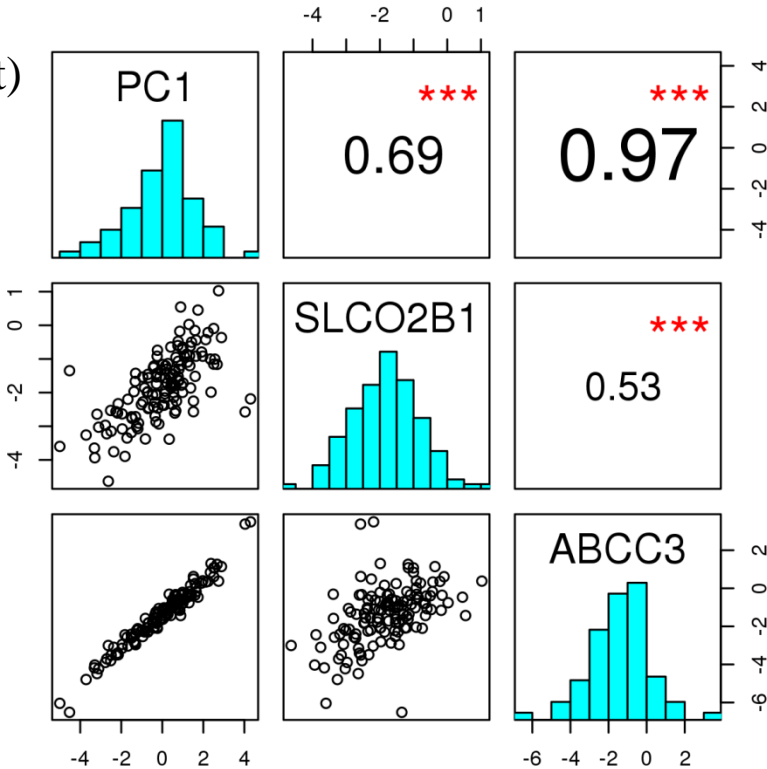

B

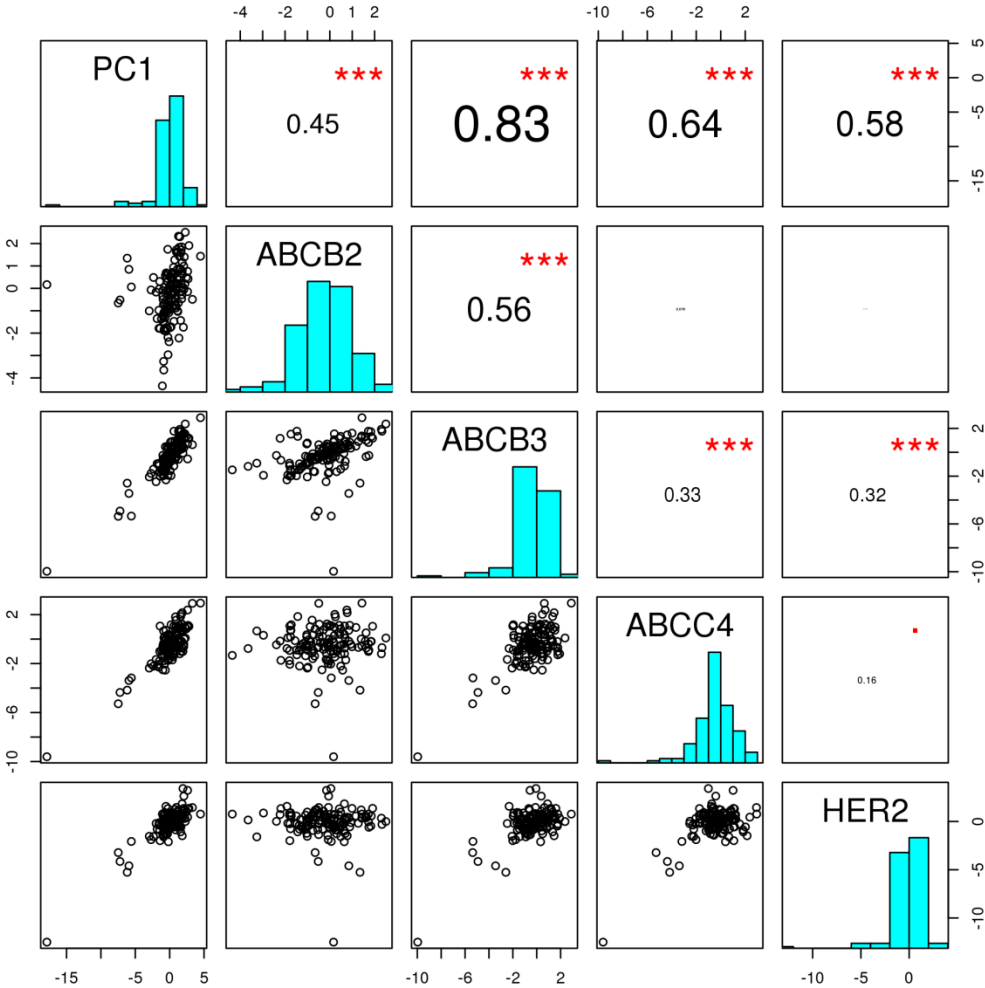

Supplement: Supplementary Figure 2 — Scatter plots showing the correlations of genes of both co-expressed networks with their first principal components (PC). Size coded values in the top-right square represent correlation coefficients and significance levels are indicated by °p < 0.1, ***p < 0.001 (Spearman's correlations). Network 1: SLCO2B1/ABCC3 (A); Network 2: ABCB2/ABCB3/ABCC4/HER-2 (B). [file Image_2.PDF]
