# Supplementary material for: Clinical Significance of Organic Anion Transporting Polypeptide Gene Expression in High-Grade Serous Ovarian Cancer
Source: Front Pharmacol. 2018 Aug 7;9:842. doi: 10.3389/fphar.2018.00842 (PMC6090214; doi:10.3389/fphar.2018.00842)
Supplement: Supplementary Table 1 — List of TaqMan® Gene Expression Assays for RT-qPCR. Assays were purchased from Applied Biosystems (Thermo Fisher, Waltham, MA) for RT-qPCR. [file Data_Sheet_1.PDF]

Supplementary Table 1: List of TaqMan® Gene Expression Assays for RT-qPCR

Gene		Assay ID		Amplicon length		NCBI Reference Sequence
SLCO1A2		Hs00245360_m1		57		NM_134431.2
SLCO1B1		Hs00272374_m1		77		NM_006446.3
*SLCO1B3**		Hs00251986_m1		69		NM_019844.3
SLCO1C1		Hs00213714_m1		92		NM_017435.3
SLCO2A1		Hs00194554_m1		87		NM_005630.1
SLCO2B1		Hs00200670_m1		113		NM_007256.2
SLCO3A1		Hs00203184_m1		89		NM_013272.2
SLCO4A1		Hs00249583_m1		80		NM_016354.3
SLCO4C1		Hs00698884_m1		77		NM_180991.4
SLCO5A1		Hs00229597_m1		60		NM_030958.1
SLCO6A1		Hs00542846_m1		63		NM_173488.3
SLCO1B7		Hs00991170_m1		87		NM_001009562.4
ABCA1		Hs01059118_m1		61		NM_005502.3.
ABCB1		Hs00184500_m1		67		NM_000927
ABCB2		Hs00388677_m1		60		NM_000593.5
ABCB3		Hs00241060_m1		66		NM_000544.3
ABCC2		Hs00166123_m1		75		NM_000392.3
ABCC3		Hs00978473_m1		57		NM_001144070.1<br>(2 transcript variants)
ABCC4		Hs00988717_m1		63		NM_001105515.1
ABCC10		Hs00375701_m1		71		NM_001198934.1
ESR1		Hs00174860_m1		62		NM_000125.3<br>(4 transcript variants)
ESR2		Hs00230957_m		163		NM_001040275.1<br>(6 transcript variants)
PXR/NR1I2		Hs01114267_m1		103		NM_033013.2;<br>(3 transcript variants)
HER-2<br>*(ERBB2)*		Hs01001580_m1		60		NM_001005862.2<br>NM_001289936.1<br>NM_001289937.1<br>NM_001289938.1<br>NM_004448.<br>(5 Ref Sequences)
PTGS2		Hs00153133_m1		75		NM_000963
HPDG		Hs00960586_g1		58		NM_000860.5<br>(5 transcript variants)
SULT1E1		Hs00960941_m1		137		NM_005420.2

Assays were purchased from Applied Biosystems (Thermo Fisher, Waltham, MA) for RT-qPCR.

For accurate normalization of mRNA between ovarian cancer tissue specimens we determined *ACTB* (PN 4326315E, Genbank Acc. No. NM_001101.2), *TOP1* (Genbank Acc. No. NM_003286), *UBC* (Genbank Acc. No. NM_021009), and *YWHAZ* (Genbank Acc. No. NM_003406) out of a panel of 12 housekeeping genes as appropriate reference genes in specimens from serous ovarian cancer (geNorm kit and geNorm software, PrimerDesign Ltd., Southampton, UK).
* Detects liver-specific *SLCO1B3* and cancer-type *SLCO1B3* variant

PTGS2: Prostaglandin-endoperoxide synthase 2 (prostaglandin G/H synthase and cyclooxygenase), *HPDG:* Hydroxyprostaglandin dehydrogenase 15-(NAD), *SULT1E1*: Sulfotransferase family 1E, estrogen-preferring, member 1
