# Supplementary material for: Clinical Significance of Organic Anion Transporting Polypeptide Gene Expression in High-Grade Serous Ovarian Cancer
Source: Front Pharmacol. 2018 Aug 7;9:842. doi: 10.3389/fphar.2018.00842 (PMC6090214; doi:10.3389/fphar.2018.00842)
Supplement: Supplementary Table 2 — Expression of SLCOs and genes coding for putative related ABC-transporters, enzymes, nuclear receptors, and HER-2 in 135 HGSOC samples. [file Data_Sheet_2.PDF]

Supplementary Table 2: Expression of *SLCOs* and genes coding for putative related ABC-transporters, enzymes, nuclear receptors, and HER-2 in 135 HGSOC samples.

	Expression	Patients	Minimum	Lower Quartile	Median	Upper Quartile	Maximum
SLCO1A2	*determined*	64	-5.37	-3.49	-2.99	-2.30	-0.21
	undetermined	71	-11.19	-9.99	-9.30	-8.79	-6.21
SLCO1B1	*determined*	2	-1.72	-1.72	-1.53	-1.33	-1.33
	undetermined	133	-7.62	-6.41	-6.02	-5.45	-2.92
SLCO1B3	*determined*	52	-6.62	-1.57	-0.69	1.17	4.53
	undetermined	83	-8.78	-7.78	-7.23	-6.74	-4.12
SLCO1C1	*determined*	4	-4.35	-3.82	-2.78	-2.25	-2.25
	undetermined	131	-10.22	-8.81	-8.33	-7.78	-5.40
SLCO1B7	*determined*	135	-7.81	-6.25	-5.61	-2.54	3.87
	undetermined	0					
SLCO2A1	*determined*	135	-6.01	-4.02	-3.20	-2.59	2.51
	undetermined	0					
SLCO2B1	*determined*	135	-4.63	-2.53	-1.77	-1.16	1.03
	undetermined	0					
SLCO3A1	*determined*	135	-3.99	-2.11	-1.46	-1.07	0.59
	undetermined	0					
SLCO4A1	*determined*	135	-6.02	-3.09	-2.15	-1.39	3.74
	undetermined	0					
SLCO4C1	*determined*	63	-5.98	-4.64	-4.07	-3.33	-1.59
	undetermined	72	-11.69	-10.17	-9.72	-9.23	-6.69
SLCO5A1	*determined*	95	-2.79	-1.14	-0.50	0.22	1.99
	undetermined	40	-9.29	-8.37	-7.95	-7.50	-5.11
SLCO6A1	*determined*	19	-8.62	-7.91	-7.29	-6.38	-5.93
	undetermined	116	-14.39	-12.79	-12.28	-11.74	-9.11
PXR	*determined*	135	-7.94	-1.73	-0.75	0.46	2.40
	undetermined	0					
ABCA1	*determined*	135	-7.92	-1.99	-0.88	0.04	5.84
	undetermined	0					
ABCB1	*determined*	135	-6.65	-4.31	-3.64	-3.02	1.53
	undetermined	0					
ABCB2	*determined*	135	-4.36	-0.98	-0.10	0.54	2.50
	undetermined	0					
ABCB3	*determined*	135	-9.97	-1.02	-0.26	0.60	2.90
	undetermined	0					
ABCC2	*determined*	135	-7.56	-5.30	-4.57	-3.88	1.11
	undetermined	0					
ABCC3	*determined*	135	-6.52	-2.20	-1.16	-0.48	3.50
	undetermined	0					
ABCC4	*determined*	135	-9.61	-1.01	-0.42	0.38	2.94
	undetermined	0					
ABCC10	*determined*	135	-8.67	-2.07	-1.08	0.06	2.78
	undetermined	0					
ESR1	*determined*	135	-6.90	-0.60	0.70	1.59	3.90
	undetermined	0					

ESR2	*determined*	135	-14.50	-8.64	-6.66	-2.88	2.88
	undetermined	0					
PTGS2	*determined*	135	-9.28	-3.58	-2.45	-1.27	2.40
	undetermined	0					
HPGD	*determined*	135	-10.75	-4.82	-2.82	-1.45	4.41
	undetermined	0					
HER2	*determined*	135	-12.50	-0.56	0.05	0.66	3.42
	undetermined	0					
SULT1E1	*determined*	135	-6.95	-4.27	-2.45	-0.55	4.71
	undetermined	0					
